# When the Red Tide Rolls In: A Red Tide Associated Angioedema Case Report

**DOI:** 10.5811/cpcem.2021.3.51920

**Published:** 2021-04-23

**Authors:** Sarah Rabinowitz, Joshua J. Solano

**Affiliations:** Charles E. Schmidt College of Medicine, Department of Emergency Medicine, Boca Raton, Florida

**Keywords:** Case report, angioedema, red tide, harmful algae, brevetoxin

## Abstract

**Introduction:**

Histamine-mediated angioedema is a potentially life-threatening reaction following exposures that incite mast cell activation. In Florida, red tides are a frequent phenomenon caused by overgrowth of the harmful algae species *Karenia brevis*, which contain environmentally detrimental brevetoxins. Even in low concentrations, brevetoxins can cause disease in humans through inducing histamine release. We report the first documented case of angioedema associated with red tide exposure.

**Case Report:**

A 52-year-old-male presented with severe angioedema encompassing both lips within a few hours after exposure to red tide algae. Other symptoms included voice changes and difficulty swallowing. Laboratory findings revealed complement factors that were within reference range, which ruled out a bradykinin-mediated pathology and supported the diagnosis of histaminergic angioedema. Symptoms resolved after 24 hours in the intensive care unit under management with epinephrine, diphenhydramine, methylprednisolone, and famotidine.

**Conclusion:**

In coastal regions, red tide algae should be recognized as a rare cause of acute angioedema. Emergency management of histamine-mediated angioedema should focus on preventing respiratory compromise with frequent airway monitoring and treatment with steroids, antihistamines, and epinephrine.

## INTRODUCTION

Angioedema is a localized, non-pitting edema of the deep dermis and submucosal tissues commonly affecting the eyelids, lips, tongue, and larynx as a result of vasodilation and increased vascular permeability mediated by histamine or bradykinin.^1^ It can be acquired, hereditary, or idiopathic and have both acute and chronic presentations.^1^,^2^ Emergency departments (ED) in the United States treat over 100,000 cases of angioedema annually.^1^,^3^ Of the different subtypes, acute histamine-mediated angioedema following allergic exposure is the most common, comprising 40–70% of all cases.^1^,^2^ There are also various environmental factors that can induce histamine release and cause angioedema.^2^

In Florida, blooms of the red tide algae *Karenia brevis* mainly occur along the Gulf of Mexico, affecting the southwest and northwest coasts of Florida.^4^ The algae blooms in response to increased macro-nutrients in coastal waters, and contributing factors include both natural ecological processes and artificial sources, such as sewage, industrial waste, and land runoffs.^4^,^5^ Red tides are considered “harmful algae blooms,” as exposure is highly toxic to marine life.^4^ Historically, significant blooms occurred infrequently; however, the Florida Fish and Wildlife Conservation (FWC) Research Institute has reported blooms with high concentrations of *K. brevis* annually since 2014 along the west coast of Florida.^6^ To date, there had been no known documented cases of angioedema induced by red tide exposure. We report one of the first cases of histamine-mediated angioedema occurring after exposure to red tide algae during a rare, east coast bloom.

## CASE REPORT

A 52-year-old man with a past medical history of gastroesophageal reflux disease, chronic pancreatitis, chronic pain syndrome, hypertension, anxiety and depression presented to the ED for evaluation of worsening swelling in his upper and lower lips onset three hours prior to arrival. His symptoms began with spontaneous right lower lip swelling that quickly progressed to encompass both lips and caused mild voice changes. Review of systems was negative for difficulty breathing, difficulty swallowing, rashes, nausea, vomiting, diarrhea, and abdominal pain.

The patient denied personal and family history of angioedema reactions, prior red tide exposure, known food or drug allergies, seafood or nut intake, and new medication exposure. He reported no changes to his medication regimen and endorsed compliance with his bupropion, pantoprazole, and methadone. The patient had started taking vitamins B12 and D regularly two days prior and had taken these vitamins in the past without issue. He did not recall any insect bites or stings and had been desensitized to bee stings in childhood. The patient’s only notable exposure was to an outbreak of red tide algae at a Palm Beach County beach that morning just prior to symptom onset.

Vital signs showed that the patient was afebrile with a blood pressure of 163/119 millimeters mercury, heart rate of 68 beats per minute, respiratory rate of 15 breaths per minute, and pulse oximetry of 94% on room air. Physical exam was notable only for isolated, severe bilateral lip edema (Image) not involving the soft palate, tongue, and uvula.

The patient was placed on two liters per minute of oxygen via nasal cannula, which improved his oxygen saturation to 96%. Initial doses of diphenhydramine, methylprednisolone, and famotidine were administered with no improvement. While still in the ED, his edema progressed, and the patient began to experience difficulty swallowing. Intramuscular epinephrine was then administered with only mild improvement two hours later. He was diagnosed with angioedema of unclear etiology and admitted to the intensive care unit (ICU) for further management. Labs were significant for leukocytosis at a white blood cell count of 10.3 thousand cells per microliter (thousand/mcL) (reference range: 5.0–10.0 thousand/mcL) with increased neutrophils. Immunology/serology showed no abnormalities: C4 complement level of 37 milligrams per deciliter (mg/dL) (16–38 mg/dL), C1q Qn of 12.3 mg/dL (11.8–23.8 mg/dL), C1 esterase inhibitor 39 mg/dL (8–40 mg/dL) and C1 ester inhibitor function of 111 (reference range: greater than 67).

**CPC-EM Capsule**

What do we already know about this clinical entity?

*Angioedema is an inflammation of the deeper dermis and subcutaneous tissues. It can be hereditary or acquired by exposure to allergens, toxins, or physical insults*

What makes this presentation of disease reportable?

*While red tides are known to cause respiratory disease, this is the first reported case of histaminergic angioedema associated with red tide algae exposure.*

What is the major learning point?

*Exposure to brevetoxins released from red tide algae may trigger angioedema reactions through mast cell activation and histamine release.*

How might this improve emergency medicine practice?

*Clinicians should be aware of local environmental triggers of histaminergic angioedema to rapidly identify and initiate appropriate treatment.*

In the ICU, the angioedema improved after additional doses of diphenhydramine, methylprednisolone, and famotidine. He did not require higher oxygen supplementation or intubation. Repeat complete blood count in the morning showed resolution of the leukocytosis. With the angioedema subsided, the patient had no difficulty breathing with an oxygen saturation of 98% on room air. He felt well enough the next day for discharge and did not experience symptom recurrence over two years.

## DISCUSSION

Brevetoxins are lipophilic neurotoxins found in the red tide algae *K. brevis*.^5^ Toxic exposure causing illness in humans mainly occurs through toxin inhalation and consumption of contaminated water and shellfish.^4^,^5^ Brevetoxins become aerosolized when the force of crashing waves lyses the cells of the *K. brevis*.^4^ The brevetoxin aerosols carried by the wind can travel up to a mile inland from their source.^7^ Their lipid solubility and small particle size enable them to penetrate skin, mucosa, and cell membranes and travel through the respiratory tract.^8^ In animal model studies, it has been demonstrated that once inside the body brevetoxins are able to disrupt voltage-gated sodium channels, causing an influx of sodium and subsequent depolarization with acetylcholine release.^8^,^9^ Consequently, this can trigger mast cell degranulation, cell apoptosis, and induce the release of inflammatory cytokines and histamine.^8^

While there are no documented cases of angioedema induced by red tides, numerous reports exist of respiratory, gastrointestinal, and neurological illnesses caused by brevetoxins.^4^,^5^,^8^,^9^ Studies previously found that red tide blooms are positively correlated with an increased incidence of ED visits for asthma exacerbations and respiratory illnesses in coastal areas.^5^,^8^,^10^

Our patient came in with severe lip angioedema of unclear etiology. His only significant novel exposure was to the outbreak of red tide algae at the beach on the same morning he developed symptoms. The October 2018 red tide bloom to which our patient had been exposed was the first red tide outbreak to affect the east coast of Florida in the last decade.^6^,^11^ According to data collected by the research institute of the Florida Fish and Wildlife Conservation Commission (Figure), the patient may have been exposed to medium to low concentrations of *K. brevis*.^6^,^11^ The presence of *K. brevis* in any concentration has the potential to cause symptoms in humans.^5^–^71^

The patient’s symptoms developed rapidly after a brief exposure and subsided within 24 hours, a clinical picture consistent with other reports of brevetoxin-induced illness, as well as non-urticarial histaminergic angioedema.^1^,^2^,^8^,^9^ The pathophysiology of angioedema and brevetoxin-induced illness described in the literature suggests a potential link, as both involve direct mast cell activation with histamine release and follow similar disease timeline.^2^,^8^,^9^

Acute histaminergic angioedema is the most common form of angioedema.^1^ One subtype of histaminergic angioedema is allergic angioedema, which often occurs after exposure to allergens or environmental triggers.^1^,^2^ It follows a type 1 hypersensitivity reaction process with immunoglobulin E (IgE) as the mediator of histamine release from mast cells and basophils.^2^ Less commonly, a non-allergic, non-IgE mediated form of histaminergic angioedema results from the direct activation of mast cells following exposure to similar triggers, physical stimuli, drugs, infections, and idiopathic events.^2^ Aside from the mechanism of mast cell activation, non-allergic and allergic histaminergic angioedema involve the same inflammatory reactions and clinical manifestations.^2^,^12^

Clinically, histamine-mediated angioedema presents rapidly within 60 minutes of an inciting exposure and resolves 24–48 hours later.^1^,^2^ Similar to anaphylactic reactions, symptoms include hypotension, tachycardia, urticaria, flushing, pruritus, bronchospasm, wheezing, laryngeal edema, nausea, vomiting, and abdominal pain.^1^,^2^ Airway compromise from laryngeal edema can manifest as stridor, voice changes, and difficulty swallowing.^1^,^2^ It is important to note that both pathways of histaminergic angioedema can present with or without urticaria and, therefore, absence of urticaria does not exclude the diagnosis.^12^ The diagnosis is clinical.^1^,^2^ Patients are often hemodynamically stable, but the systemic vasodilation can induce hypovolemic shock and respiratory distress.^2^

Routine workup may show leukocytosis, as seen in our case.^1^ Acute histamine-mediated angioedema can have normal or elevated serum tryptase levels, but this is not routinely obtained.^1^ Otherwise, in the ED setting there are no specific laboratory findings that will indicate the diagnosis and guide management. Unlike in bradykinin-mediated angioedema, the levels and function of complement proteins (C4, C1q, C1 esterase inhibitor) are normal in histamine-mediated angioedema.^1^,^2^,^12^ Management involves airway preservation, epinephrine, steroids, and histamine H1/ H2 receptor blockers.^1^,^2^ In the ED setting, improvement of the angioedema in response to these treatments supports the diagnosis of histamine-mediated angioedema, even in cases without obvious urticaria or allergic manifestations.^1^,^2^

This is essential to recognize, as bradykinin-mediated angioedema subtypes follow along different pathways involving complement factor deficiencies; thus, they do not respond to treatment with steroids, epinephrine, and antihistamines, have higher rates of reoccurrence, and worse clinical outcomes.^1^,^2^ Therefore, early identification of a histaminergic process is crucial for emergency management, as treatment of bradykinin-mediated angioedema focuses on correcting the underlying completement deficiencies.^1^ Delays in initiating appropriate therapeutic intervention can become disastrous in the setting of worsening shock and respiratory compromise.^1^,^2^

Lastly, it is important to address the likelihood of the patient’s medications as underlying causes of his angioedema. There have been several case reports of urticarial angioedema occurring within the first four weeks after initiating bupropion therapy.^13^,^14^ Unlike the cases described in the literature, our patient had been taking a consistent dose of bupropion for longer than four weeks. Furthermore, his bupropion was continued throughout admission with resolution of his angioedema, which supports that his angioedema process was most likely unrelated to his bupropion exposure. Likewise, hypersensitivity reactions to opiates, proton pump inhibitors, and vitamin capsule ingredients have been occasionally cited as possible triggers causing urticaria and angioedema.^2^,^15^ However, complete resolution of symptoms would be dependent on discontinuing exposure to the substance.^2^,^15^ Our patient’s pantoprazole was also continued throughout admission, with methadone and vitamins resumed prior to discharge without issue. Re-exposure to these substances would have resulted in another episode of angioedema; therefore, it is unlikely that these medications were the cause.

## LIMITATIONS

The patient did not clarify the nature and duration of his red tide exposure. Therefore, it is unclear whether he had gone into the water and was still at the beach when his symptoms began.

## CONCLUSION

Acute histamine-mediated angioedema is a common yet potentially fatal edematous reaction to triggers of mast cell degranulation and histamine release. Early recognition of a histamine-mediated etiology is essential for both acute treatment and long-term management, which depends on avoiding the inciting event. We present the first documented case of red tide-associated angioedema. In coastal areas where red tide blooms are common, it is important to consider *K. brevis* brevetoxins as a possible etiology in a patient presenting with acute onset of angioedema.

## Figures and Tables

**Figure:**
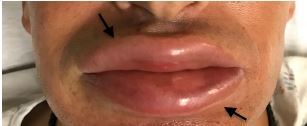
Map showing the statewide *Karenia brevis* concentrations October 1–31, 2018. Location of patient’s red tide exposure (white arrow). Reprinted with permission from the research institute of the Florida Fish and Wildlife Conservation Commission.^11^

**Image:**
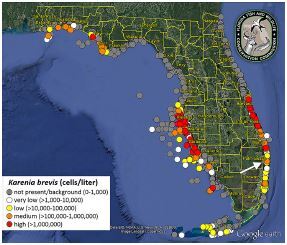
Impressive angioedema diffusely encompassing both lips (arrows).

## References

[1] Long BJ, Koyfman A, Gottlieb M (2019). Evaluation and management of angioedema in the emergency department. West J Emerg Med.

[2] Busse PJ, Smith T (2017). Histaminergic angioedema. Immunol Allergy Clin North Am.

[3] Kelly M, Donnelly JP, McAnnally JR (2013). National estimates of emergency department visits for angioedema and allergic reactions in the United States. Allergy Asthma Proc.

[4] Patel SS, Lovko VJ, Lockey RF (2020). Red tide: overview and clinical manifestations. J Allergy Clin Immunol Pract.

[5] Hoagland P, Jin D, Beet A (2014). The human health effects of Florida red tide (FRT) blooms: an expanded analysis. Environ Int.

[6] Florida Fish and Wildlife Conservation Commission Red Tide.

[7] Cheng YS, Zhou Y, Pierce RH (2010). Characterization of Florida red tide aerosol and the temporal profile of aerosol concentration. Toxicon.

[8] Fleming LE, Kirkpatrick B, Backer LC (2011). Review of Florida red tide and human health effects. Harmful Algae.

[9] Abraham WM, Bourdelais AJ, Sabater JR (2005). Airway responses to aerosolized brevetoxins in an animal model of asthma. Am J Respir Crit Care Med.

[10] Kirkpatrick B, Fleming LE, Backer LC (2006). Environmental exposures to Florida red tides: effects on emergency room respiratory diagnoses admissions. Harmful Algae.

[11] (2018). FWC Fish and Wildlife Research Institute Statewide *Karenia brevis* Concentrations.

[12] Wu MA, Perego F, Zanichelli A (2016). Angioedema phenotypes: disease expression and classification. Clin Rev Allergy Immunol.

[13] Tackett AE, Smith KM (2008). Bupropion-induced angioedema. Am J Health Syst Pharm.

[14] Tuman TC, Tuman BA, Göksügür N (2016). Urticaria and angioedema associated with bupropion: three cases. Prim Care Companion CNS Disord.

[15] Bowlby HA, Dickens GR (1994). Angioedema and urticaria associated with omeprazole confirmed by drug rechallenge. Pharmacotherapy.

